# Modelling Mobility of Hunter-Gatherer Populations: A Dynamic Simulation Approach Based on Cellular Automata

**DOI:** 10.1007/s10816-025-09726-4

**Published:** 2025-08-02

**Authors:** Richard J. Hewitt, Manuel Alcaraz-Castaño, Vito C. Hernandez, Mike W. Morley

**Affiliations:** 1 Instituto de Economía, Geografía y Demografía, Spanish National Research Council (IEGD-CSIC), C/de Albasanz 26-8, Madrid, 28037 Spain; 2 Área de Prehistoria (Departamento de Historia y Filosofía), Universidad de Alcalá, Colegios 2, Alcalá de Henares, 28801 Spain; 3 Flinders Microarchaeology Laboratory, College of Humanities, Arts and Social Sciences, Flinders University, South Australia, Australia

**Keywords:** Cellular automata, Hunter-gatherer mobility, Palaeolithic Iberia, Agent-based model, Decision catchment

## Abstract

Understanding mobility of past hunter-gatherer populations requires dynamic approaches which incorporate uncertainty. Least cost models assume complete knowledge of the terrain on the part of the traveller, while ethnographic examples tend to be specific to the groups and territories studied. Most least cost models also assume that origin points, destination points, or both, are known in advance, limiting their utility for exploring movement potential in landscapes where evidence for occupation is scarce. This research addresses these limitations through an agent-based model of movement grounded in cellular automata (CA) theory, called DISPERSCA. Agents depart from a point, which may be specified or determined at random, and transit a fitness landscape for a fixed number of iterations according to decisions made within a defined area at each time step (*a decision catchment*), the CA neighbourhood. If the decision catchment is unknown multiple runs are made at different CA neighbourhood sizes and the results are compared. Neighbourhoods may be square or hexagonal, the former producing on average longer displacements, the latter ensuring that individual walks are of equal length in any direction. The model is demonstrated by application to Late Pleistocene Central Iberia, where confirmed archaeological sites are scarce. Some support can be advanced for the hypothesis that the Central Iberian mountains, probably combined with the Iberian System range, presented a significant barrier to hunter-gatherer groups. The model can be modified to account for agents’ prior knowledge, or to include fitness variables unrelated to terrain cost, such as water, the presence of game animals or vegetation.

## Introduction

Understanding the mobility of past hunter-gatherer populations in and around the landscapes they occupied is an enduring challenge for archaeologists and anthropologists (*e.g.* Binford, 1980; Whallon, 2006; Perreault & Bratingham, 2011; Kelly, 2013). For example, researchers may seek to identify possible relationships and likelihood of range overlap between different species or groups (*e.g.* Neanderthals and early *Homo sapiens*) or explore how hunter-gatherer populations might have exploited key food resources or raw material for lithic manufacturing (Aubry *et al*., 2012; Picin, 2022; Vaissié *et al*., 2021).

However, many approaches to understanding mobility in past populations seek to derive insights from static analyses of idealised characteristics of the territory, such as least cost routes or time–cost catchment zones, or descriptive comparisons with ethnographic examples, which often overlook the wide diversity of hunter-gatherer lifeways (Kelly, 2013) and the difficulties of categorising their mobility strategies (Perreault & Bratingham, 2011). Few studies employ dynamic approaches to model the movement of hunter-gatherer agents as they cross the landscape (though see Brantingham, 2003; Gravel-Miguel & Wren, 2018; Garg *et al*., 2021). As a result, existing literature sheds little light on the diversity and type of possible interactions between hunter-gatherers and their resources in space and time in a given territory.

In this paper, we propose a simple agent-based model grounded in cellular automata theory to address this research gap. First, we discuss and review relevant literature for mapping and modelling movement in general (‘Research Background’ section). Next, we provide the theoretical and mathematical basis for the model and describe in detail its specification and operation (‘Methods’ section). Subsequently, we apply the model to illuminate the exploration and transit of a mountainous barrier by mobile hunter-gatherer groups for the case of Palaeolithic Iberia (‘Results’ section). We conclude with an extended discussion of how these types of models can inform us and suggest other possible avenues for their application (‘Discussion and Conclusions’ section).

## Research Background

### Least-Cost Paths and Catchments

Researchers have sought to understand hunter-gatherer mobility from many different perspectives and scales. Some approaches, like the wave of advance model (Ammerman & Cavalli-Sforza, 1979) or the site catchment modelling approaches of Vita-Finzi *et al*. (1970) predate the application of quantitative methods using modern computers. Others are closely allied to the emergence of geographical information systems (GIS) desktop software in the 1980 s and 1990s. For example, there is a large body of work around the concept of ‘cost’, which concerns the relative difficulty of transit across regions, resource locations or occupation sites, on the basis of physical characteristics like terrain slope, vegetation cover, or ocean currents. Estimating the easiest (and assumed most probable) lines of transit or Least Cost Paths (LCPs) is one of the oldest applications of GIS in archaeology and has evolved to become a standard operation undertaken in many contexts and periods. These include exploring the location of Roman roads (Güimil-Fariña & Parcero-Oubiña, 2015), understanding connections and tribal territoriality associated with agriculture (Howey, 2007), mapping the distribution of Palaeolithic symbolic objects (Gravel Miguel & Wren, 2018), or reconstructing historical journeys (Seifried & Gardner, 2019).

LCPs are frequently used in models of hunter-gather mobility, resource exploitation or social interaction (*e.g.* Rissetto, 2012; Moreno-Maynard *et al*., 2022; Pallo and Borazzo, 2016). Many studies assume complete knowledge of the territory by the groups investigated, which seems questionable except for small areas. While ethnographic parallels suggest that hunter-gatherer groups were often highly mobile across large land areas (trips in excess of 250 km; *e.g.* Loebel, 2005, Ellis, 2011), returning seasonally to exploit the same places, detailed knowledge of such large areas seems unlikely to extend to microtopography down to a few metres. It is more probable that groups, over time, would have acquired knowledge of the general characteristics of their territory. A further limitation, common to many studies of hunter-gather mobility using LCPs, is a general assumption of homogenous groups moving across an unvarying landscape. Such approaches may fail to account for resting, subsistence, differences between hours of light and dark, seasons, weather or surface conditions, opportunistic detours, or travelling in family groups with individuals of differing physical aptitude.

Conventional LCP calculations tend to assume knowledge of the best route in advance of travel. This is clearly a plausible assumption where there is only one clearly viable route, as in the case of a mountain pass, where distances travelled are assumed to be short, or where it can be assumed that the groups in question have made the trip before. But the conventional LCP model breaks down where there are many possible alternative routes with only minor differences in cost, where distances travelled are longer, or where journeys are assumed to be pioneering or exploratory. It seems unrealistic to suppose that groups would travel many hundreds of kilometres along strict LCPs, especially when points of attraction, like food sources, water, or raw materials are located at intermediate points.

Some studies advance these simple methods in rewarding directions. For example, Gravel-Miguel & Wren (2018) employ an agent-based LCP model in which agents take least cost decisions at each time step based on locally observable surroundings. Different parameter options can be chosen based on whether the agent is exploring or trying to reach the goal as quickly as possible, and a stochastic parameter is included to allow the agent to choose between different points with identical cost. The model generates many possible LCP allowing zones of high variability—many possible routes—to be compared with mobility bottlenecks, where there are few suitable routes.

Lewis (2021) draws attention to the often-ignored problem of error in the source elevation data. To try to circumvent this issue, they develop a least cost model which randomly distributes specified root mean square error (RMSE) across the digital elevation model surface (DEM) over multiple runs, thus generating multiple LCPs with minor variations (Monte Carlo simulation). By aggregating the multiple model runs the most probable routes can be identified while accounting for error (Lewis, 2021).

In order to address the problem that different approaches to LCP tend to give different results, Jiménez *et al*. (2024) apply three techniques: LCP calculation based on terrain slope, LCP calculation based on the well-known ‘hiking function’ of Waldo Tobler (Tobler, 1993; White, 2015), and a unit cost approach, in which a cost value is calculated for each cell in the map representing the effort required to cross it (Prieto *et al*., 2016). Llobera & Sluckin (2007) address the well-known problem of differences in cost between uphill and downhill travel (anisotropy) using a metabolic curve which accounts for slope direction as well as additional energy expenditure required for changing direction.

An extension of the LCP approach, which would seem to recognise the somewhat unrealistic nature of exact LCP calculations as an approximation of hunter-gatherer mobility, is the calculation of time-dependent catchment areas, *i.e.* buffer zones or consecutive time rings expressing spatially the approximate travel time required to reach the centre of each zone (see *e.g.* Byrd *et al*., 2016, Vaissié, 2021).

Finally, the oft-cited limitation that LCPs require two known points is overcome by White & Barber’s (2012) From Everywhere to Everywhere (FETE) model, in which every possible connection to a set of points is calculated, enabling the identification of key routes connecting the greatest number of nodes in the network. This approach has been applied to great effect by Bilotti *et al*. (2024), in a highly innovative study of Mediterranean trade networks linking both marine and land-based communication networks in a single model. Crabtree *et al*. (2021) use the FETE model, supported by high-performance computing, to identify optimal migration pathways, or ‘superhighways’ across the continent of Sahul (the connected land masses of Australia and New Guinea), which they hypothesise would have facilitated expansion and colonisation of the continent during the later Pleistocene.

### Modelling Movement

Beyond the question of least cost paths and time-distance catchments, several studies seek to reconstruct the immediate environment of hunter-gatherer occupation sites to understand how past populations might have used their available resources. These studies pinpoint specific locations such as outcrops where raw material was sourced (*e.g.* Aubry *et al*., 2012, Picin, 2022, Jiménez *et al*., 2024, *inter alia*) and seek to shed light on the types and range of vegetation potentially available at the date of occupation (*e.g.* Hughes *et al*., 2007, Jones, 2013). However, the pattern of mobility in and around occupation sites is hard to determine from such studies alone. Palaeoenvironmental reconstruction can determine to some extent the available range of vegetation, and suggest, for example, whether environments were closed or open, with implications for mobility patterns (Tougard & Montuire, 2006; McAllister-Hayward *et al*., 2024). However, approximating realistic movement patterns requires more dynamic approaches, *e.g.* by modelling the simulated movements of virtual populations exploring the characteristics of the actual or reconstructed landscape in space and time. This is the approach we take in this paper.

In the work described in this paper, we build on these earlier studies and innovate by moving in several new directions. Firstly, we apply an agent-based approach which models individual movement in the landscape as arising from multiple decisions under uncertainty, accounting for incomplete knowledge on the part of hunter-gatherer agents. In Gravel-Miguel and Wren’s model, incomplete knowledge is incorporated by accounting for local visibility at each time step, and by allowing agents to make random decisions where route options are identical (Gravel-Miguel & Wren, 2018). In our model, we address this by introducing cellular automata (CA) theory (see *e.g.* Itami, 1994), such that agents’ location choices are determined at each time step by computing a transition function from cost values in the agents’ cell neighbourhood. We propose the concept of a *decision catchment*, which may vary depending on the type (hexagonal or square) and size of the neighbourhood. The size of the neighbourhood is a key point of discussion in geographical CA applications, as it relates directly to the scale of at which processes are modelled (Kocabas & Dragicevic, 2006, Barreira-González & Barros, 2017, Roodposhti *et al*., 2020, Ménard & Marceau 2005, Díaz-Pacheco *et al*., 2018). Since the CA neighbourhood aggregates the cost from the underlying raster, our decision catchment approach is less sensitive to raster cell resolution of the base data, particularly where neighbourhood cell size is large. We also incorporate randomness in the same way as Gravel-Miguel & Wren (2018). Theoretically, CA moves beyond earlier concepts of resource catchments like central place foraging models (Byrd *et al*., 2016), towards a more dynamic understanding of the territory, in which time and space are experienced by individuals as constraining or enabling factors (LLobera, 2000).

Most importantly, while we demonstrate our model with a simple slope map and define least cost following the previously mentioned traditional focus of energy expenditure moving across terrain, the model is not in any sense restricted to such an application. It provides a way to simulate movement of individuals or populations, whether animals or people, within a landscape under any constraining or enabling factor that can be expressed on a map. Any variable that can be aggregated from an underlying map into the cell neighbourhood can be used to calculate the agents’ transition function at each time step, for example, distance to water, attractiveness of a known site or monument, or presence of predators or prey. In this sense, our decision catchments can also be seen as *opportunity catchments*.

## Methods

### Model Specification

The model we propose addresses the key theoretical limitations of many existing mobility models by introducing a dynamic, rather than static, conception of hunter-gatherer movement around a landscape. Rather than assuming knowledge of the best route in advance, as in conventional least cost path calculations, movements by a hunter-gatherer agent or group are made successively from one point to another, with the location of each new destination point being determined by knowledge acquired by the agent while located at the previous point. Similar to the study by Gravel-Miguel & Wren (2018), such knowledge could include the agents’ view of local terrain, as well as much more distant landscape features. However, given the possibility of visual obstructions, an alternative approach, which we propose here, would be to limit an agent’s decision-making space to a restricted neighbourhood around its immediate location. Thus conceptualised, hunter-gather movement is susceptible to the mathematical theory of cellular automata, best described in the words of one of the theory’s founders, Stanislaw Ulam, as follows:

Given is an infinite lattice or graph of points, each with a finite number of connections to certain of its “neighbours”. Each point is capable of a finite number of “states”. The states of neighbours at time t_n_ induce, in a specified manner, the state of the point at time t_n+1_. This rule of transition is fixed deterministically, or more generally, may involve partly “random” decisions. (Ulam, 1952)

**Fig. 1:**
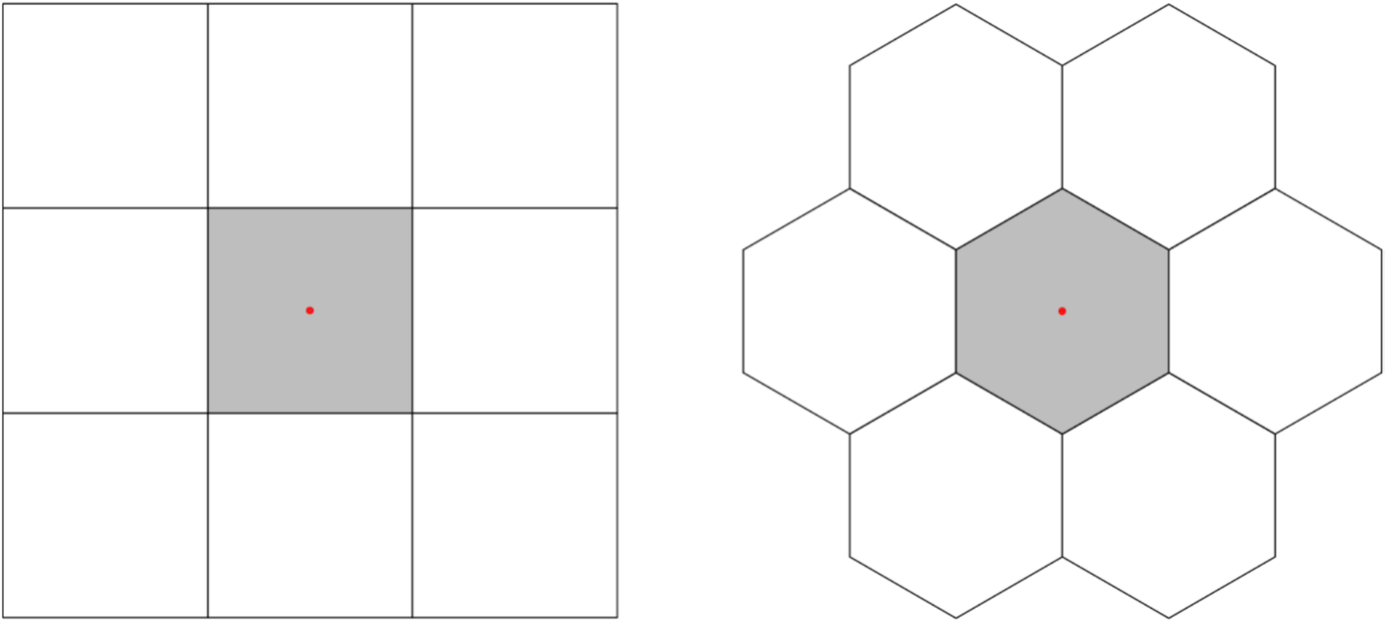
(left) A square ‘Moore’ neighbourhood, with an agent (red dot) located at its centre (grey cell); (right) the same situation, represented by a hexagonal neighbourhood. Source: own work, after Slimi & Yacoubi (2009)

The landscape across which the agent must move is our infinite lattice, analogous to a boardgame with a rectangular or hexagonal grid (Fig. 1), and the agent is situated within a single cell on the lattice, the neighbours being those directly in contact with this cell, which would logically be understood to include the corner cells (a Moore neighbourhood).

The starting point for a particular agent on the lattice can be set to a specific location or determined randomly. The neighbourhood is then defined, and the agent evaluates the values of some phenomenon in its neighbourhood. In the simplest case of a least cost model, the lattice overlies a cost surface, for example, terrain slope, and the slope values are extracted for each cell in the neighbourhood and compared, analogous to an individual or group considering the most suitable way to cross a particular patch of ground. The agent selects the cell with the minimum overall cost, and moves to that cell, around which a new neighbourhood is defined. The process is repeated for *n* iterations or until the edge of the map is reached. Previously visited locations are excluded from each neighbourhood. When the simulation ends, the points visited by the agent, and the path taken from the beginning to end of the trajectory can be plotted on screen or exported to file.

This cellular-automata model can be expressed by the following equation:

1 $$ij^{t+1}=min\left(n_{ij}^t\right)$$

where ${n}_{ij}^{t}$ is the value of the cost variable for neighbourhood *n* centred around the cell at row *i* and column *j* on the lattice which is occupied at the current time step *t,* and $i{j}^{t+1}$ is the cell at row *i* and column *j* on the lattice to be occupied at the subsequent time step *t* + 1.

**Fig. 2:**
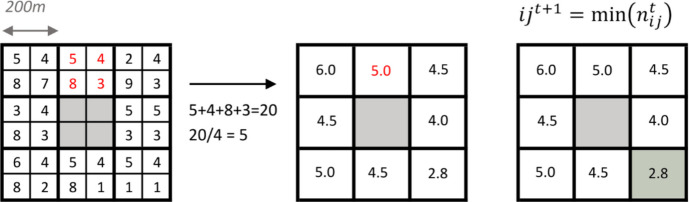
Example of a 200 m cell resolution neighbourhood overlying a 100 m cell resolution cost surface. Each of the 8 neighbourhood cells overlying the cost surface contains 4 cells from the cost surface, the mean of these 4 cells gives the cost value for the overlying cell. For the worked example (red highlighted cells), the mean of the 4 cells is 5. Finally (far right), the cell containing the minimum value (2.8) of the 8 cells in the neighbourhood is selected and the agent moves to that cell

Note that in the case that the neighbourhood cell size is larger than the underlying grid on which the cost variable is represented, each cell in the neighbourhood will have several values. In the currently described model, the mean of these values is taken for each cell in the neighbourhood before it is passed into the minimising equation (Eq. 1). Therefore:

2 $$n_{ij}^t=mean{({ni}_k)}_{k=1}^x$$

where ${n}_{ij}^{t}$ is the value of the cost variable for neighbourhood *n* centred around the cell at row *i* and column *j* on the lattice, which is occupied at the current time step *t*, and ${ni}_{k}$ is the collection of values from the raster cost surface that fall into each individual cell *ni* in the neighbourhood *n.* The total number of values included in each individual cell *ni* is denoted by* x* and depends on the resolution of the underlying raster and the size of the overlying neighbourhood cell *ni.* For example, in the case of a neighbourhood composed of cells measuring 200 × 200 m, overlying a cost surface of resolution 100 × 100 m, the cost value for each neighbourhood cell *ni* will be obtained from the mean of 4 individual values in the cost surface (*x* = 200/100 × 200/100 = 4) (Fig. 2).

Note that in the model described here, while the cell size of the neighbourhood *nsize* can be varied, only a single ring of consecutive neighbours is considered. The size of the neighbourhood is therefore determined by the size of the individual cells and represents the agents’ decision-making space. This is of key importance and can be determined by some known quantity, *e.g.* estimated size of particular agents’ territories, or, in the usual case where this is unknown, the model can be run with multiple values of the *nsize* parameter, to see which kind of patterns emerge in aggregate.

**Fig. 3:**
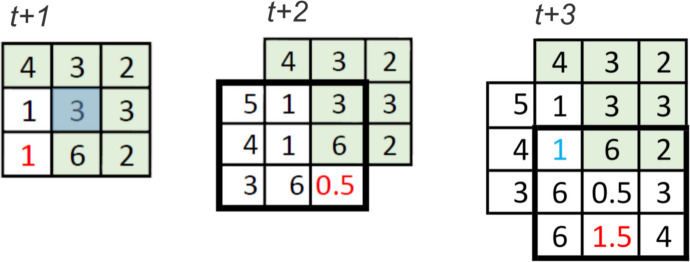
Example of three time steps; left: at time *t* + 1, Eq. 1 has already been applied to the neighbourhood centred around the starting cell (blue shaded), to obtain the minimum value in the neighbourhood (1). Since there are two minimum values, the bottom left cell has been randomly selected. The value in red indicates the new location of the agent. Centre: at time *t* + 2, Eq. 1 is applied to the new neighbourhood and the single minimum value (0.5, in red) has been chosen as the new location of the agent. Right: at time *t* + 3, Eq. 1 is applied to the new neighbourhood, resulting in selection of the lowest value (1 in blue). However, this cell corresponds to a previously visited location and is excluded from the selection set. The next minimum value in the set (1.5, in red) becomes the agent’s new location

Two final considerations are necessary to specify the working model (Fig. 3). First, where the minimum value is identical for more than one cell in the neighbourhood, one of these competing minimum cells is chosen at random as the next step in the agents’ walk. Secondly, those cells already visited must be excluded from the neighbourhood selection to avoid the model becoming trapped in a see-saw type situation where the walk oscillates back and forth between the same few cells. However, it can occasionally occur that all cells have been previously visited, in which case, the new location is chosen, as in the previous case, at random.

These two considerations mean that the model is not entirely deterministic since successive runs of the model will encounter these problems at the same points but resolve them in different ways each time as a result of the randomisation function. However, most of the time a random solution for the next walk location will be required only rarely. The use of occasional random values should not greatly affect the overall walk in any case.

The model was programmed in the R environment, and in its simplest form takes just one input, a GIS raster layer representing a cost or friction surface. While the friction surface could be a suitability layer of various composite variables affecting diffusion through the landscape (vegetation, water, *etc.*), for demonstration purposes we use just a simple slope map (in degrees) obtained from a digital terrain model. The model outputs both point and line vector GIS format files (.gpkg) of the walk undertaken for each model run.

To apply the model to a practical case, it is necessary to determine the size of the neighbourhood to be used by the hypothetical agents when taking cost decisions at each time step (*the decision catchment*). A priori, we do not know what the most appropriate catchment for hunter-gatherer groups or individuals moving around this landscape is likely to be, and nor would be expect it to be a constant size. Rather than simply assigning an arbitrary value, one solution would be to choose a catchment based on ethnographic proxies. The disadvantage of such an approach is that these kinds of data tend to relate to specific groups carrying out specific activities at specific times in specific landscapes (*e.g.* Loebel, 2005, Ellis, 2011). An alternative solution to the problem would be to run the model for several different catchment sizes in order to examine the difference between them. Such an approach makes explicit the dependency of the model on the chosen catchment size and allows the difference between catchments of different sizes to be clearly explored.

### Demonstration Case Study Area

**Fig. 4:**
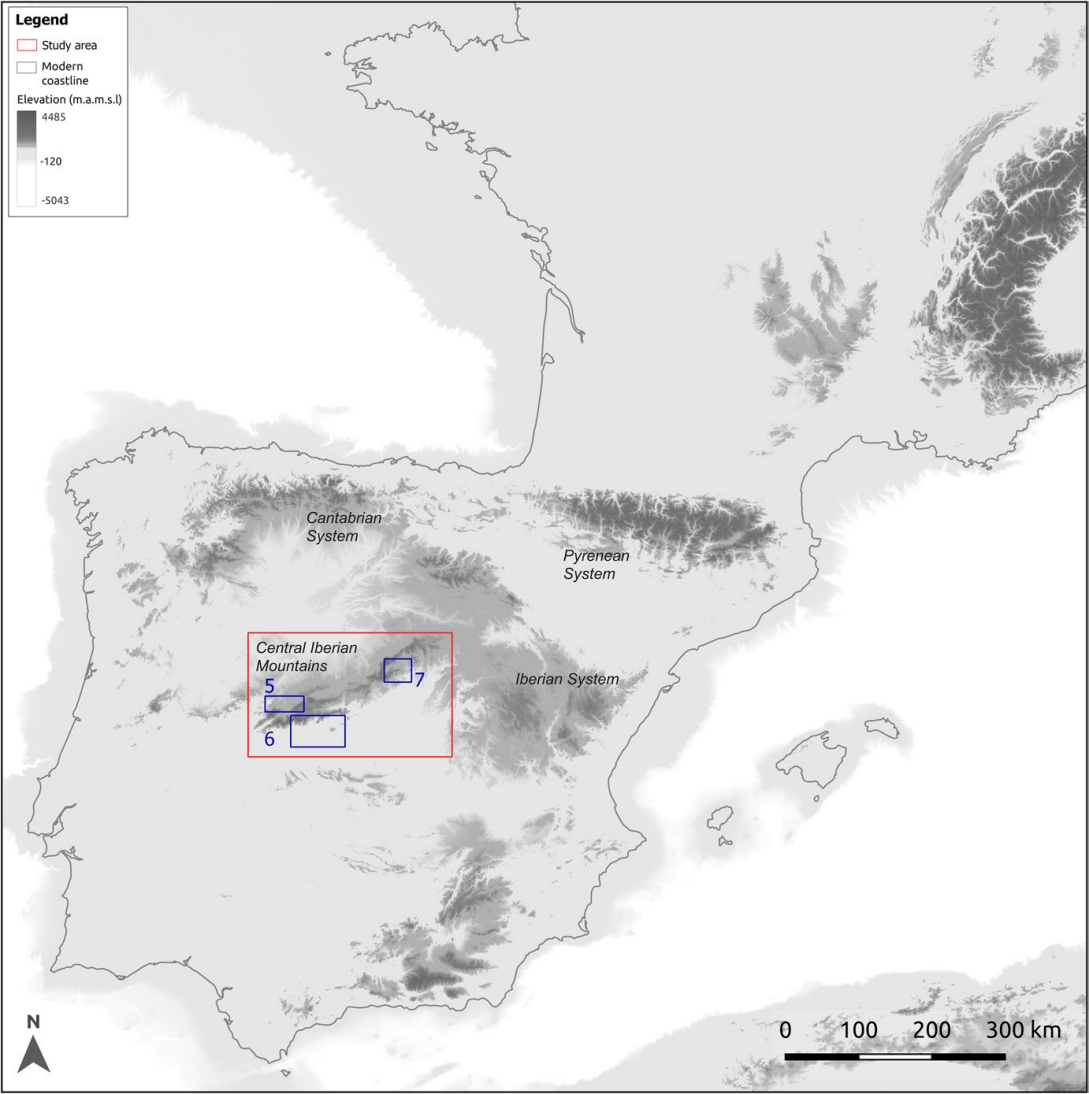
Southwest Europe, showing the study area and mountain ranges of Iberia referred to in the text. Light grey shading beyond the modern coastline indicates the coastline at the Last Glacial Maximum (LGM) ~ 23 and 19 ka cal BP (Cascalheira *et al*., 2021), 120 m below modern mean sea level. Blue inset boxes within the study area refer to walks depicted in Figs. 5, 6 and 7

We demonstrate the application of our model, which we call DISPERSCA, for exploratory analysis of potential hunter-gather mobility across the whole of the Central Iberian Mountain range (in present-day Spain), an area of approximately 48,000 km^2^ (Fig. 4). The choice of study area was motivated by the active ongoing debate around the dispersion of human populations in Iberia during the Middle and Upper Palaeolithic. For years, the distribution of Late Middle Palaeolithic and Early Upper Palaeolithic sites in Iberia, with an absence of the first phases of the Aurignacian technocomplex in the centre and south of the peninsula and the potential late survival of the Mousterian in these territories, have been intensively discussed (*e.g.* Haws *et al*., 2020; Alcaraz-Castaño *et al*., 2021; Zilhão, 2021). Explanations for this odd population pattern, and more broadly for understanding the Neanderthal demise and the first modern human settlement of Iberia, have been increasingly developed in recent years. Among these, simulation modelling approaches figure prominently, *e.g.* those based on environmental and biogeographic constraints, including climate variability (Burke *et al*., 2017; Klein *et al*., 2023; Shao *et al*., 2024) and related factors such as ecosystem productivity (Vidal-Cordasco *et al*., 2022) or herbivore carrying capacity (Vidal-Cordasco *et al*., 2023). In addition, the well-known ‘Ebro Frontier’ model seeks to explain the potential late persistence of Neandertals and late arrival of modern humans to the centre and southern regions of Iberia. Proposed by J. Zilhão in the 1990’s and recently revised, this model postulated the difficulty of traversing the mountainous regions of Iberia due to the significant north–south barriers presented by the Cantabrian, Pyrenean, and Iberian ranges (Zilhão, 2021). Here, we focus on the more southerly Central System range to explore human mobility patterns across these mountains. Thus, while our principal aim is to demonstrate our model’s utility and potential for modelling hunter-gatherer mobility in general terms, we also look to shed light on this key research question: assuming similar topographic relief in the Central System mountain range at least since the last glacial retreat around 19–20 kya (Oliva *et al*., 2019), how might hunter-gatherer groups have negotiated this hypothetical mountainous barrier?

**Fig. 5:**
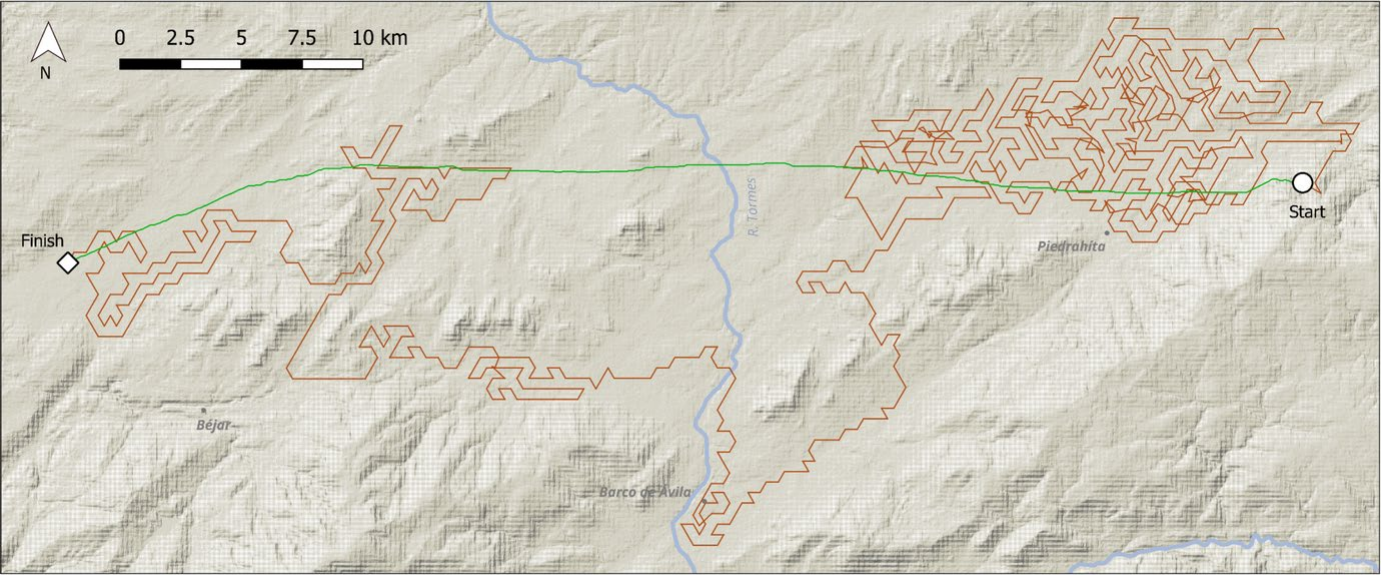
Walk example 1, 500 m neighbourhoods. River names and modern place names included for orientation. The end points specify the final point reached after 1000 timesteps. The walk remains for many iterations in a low-lying plain known as the Amblés valley, east of modern-day Piedrahíta. The green line indicates the anisotropic least cost path, calculated by the GRASS GIS module *r.walk*

**Fig. 6:**
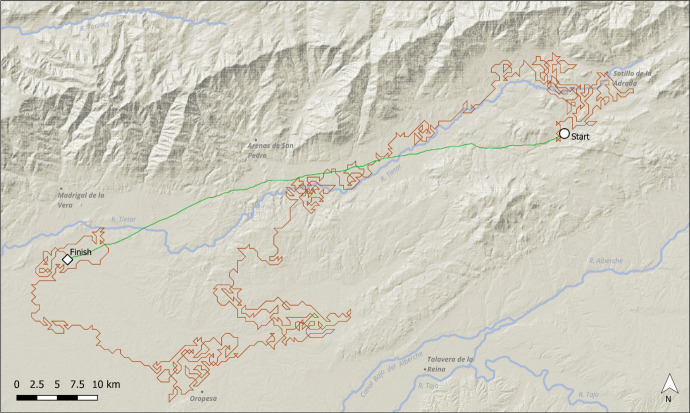
Walk example 2, 500 m neighbourhoods. River names and modern place names included for orientation. The end points specify the final point reached after 1000 timesteps. In this example, the walk carefully avoids the Gredos range to the north, preferring to head south-east following the present-day River Tietar. The green line indicates the anisotropic least cost path, calculated by the GRASS GIS module *r.walk*

**Fig. 7:**
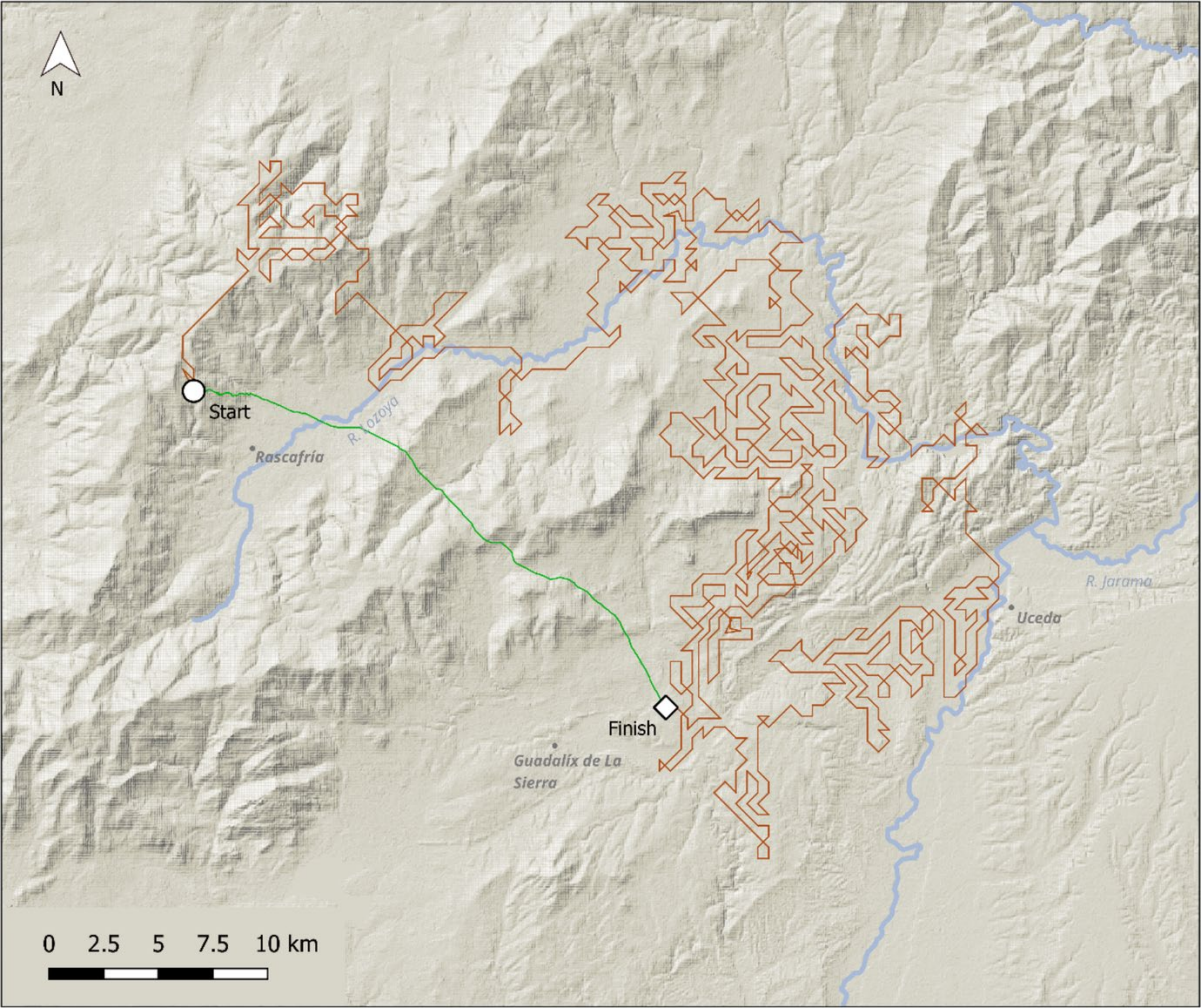
Walk example 3, 500 m neighbourhoods. In this example, the walk begins on the southern slopes of the Guadarrama range, then descends following the Lozoya valley to the south. The green line indicates the anisotropic least cost path, calculated by the GRASS GIS module *r.walk*

To address this research question, we use the model to explore the ways groups might have moved around, and crossed, the central ranges, and whether or not certain locations are likely to have been particularly favoured. To execute the model, an agent, representing a hunter-gather group, was located randomly within the study area of Central Iberia, from which point its walk was determined by the least cost decisions it took in its neighbourhood. The agent walked for 1000 timesteps or until the edge of the map was reached. The process was repeated 100 times for each model configuration corresponding to the agents’ least cost decision catchment of 1 km, 500 m, and 100 m, respectively, and with both square and hexagonal cell neighbourhoods. Aggregate results raster layers were created with cells allocated a value of 1 every time they were crossed by a least cost walk. In this way, the parts of each study area most frequently crossed by the individual walks could be identified by visualising the highest value cells (most frequently crossed) in darker colours.

Total distances (the accumulated distance travelled by all walks in a single run) and resultant distances (the straight-line distance from start to finish for a single run) were calculated and exported to a separate file. The difference between total walk distances and resultant distances was calculated to estimate *walk deviation*—the larger the difference, the greater the deviation from the average direction of the individual walk (Allen *et al*., 2015). The difference was expressed as a percentage of the total walk distance, in order to enable comparison between runs of different neighbourhood sizes, with correspondingly different walk distances.

Finally, azimuth bearings (calculated following the quadrant method, see Uren & Price 1994, p. 14–15) were computed from the resultants, indicating the compass direction of each 1000 step walk. The azimuth bearings were used to plot square root-scaled orientation rose diagrams (see Allen *et al*., 2015, Hewitt *et al*., 2018), with the aim of detecting preferential orientation of the walks. To test for preferential orientation well-known statistical tests for circular data were used, the Rayleigh Test and Kuiper’s Test (see Hewitt *et al*., 2018).

## Results

As would be expected from a randomly-located selection of starting points, many walks stayed to one side of the Central Iberian Mountain range (Fig. 4) and did not cross it, repeatedly exploring low-cost troughs corresponding to localised plateaus surrounded by higher ground, or low-lying land beyond the mountain slopes (Fig. 5). Other walks found paths that led along the ranges for many kilometres, hugging the shallower slopes of the foothills without moving to steeper ground at all (Fig. 6). However, a number of walks did cross the ranges from south to north or *vice-versa* (Fig. 7) sometimes incompletely. Though the routes taken were often circuitous, they corresponded to well-known mountain passes, like the valley of the river Alberche, in between the Guadarrama and Gredos ranges, or the rivers Lozoya and Jarama, in the northern Guadarrama range (Fig. 7).

**Fig. 8:**
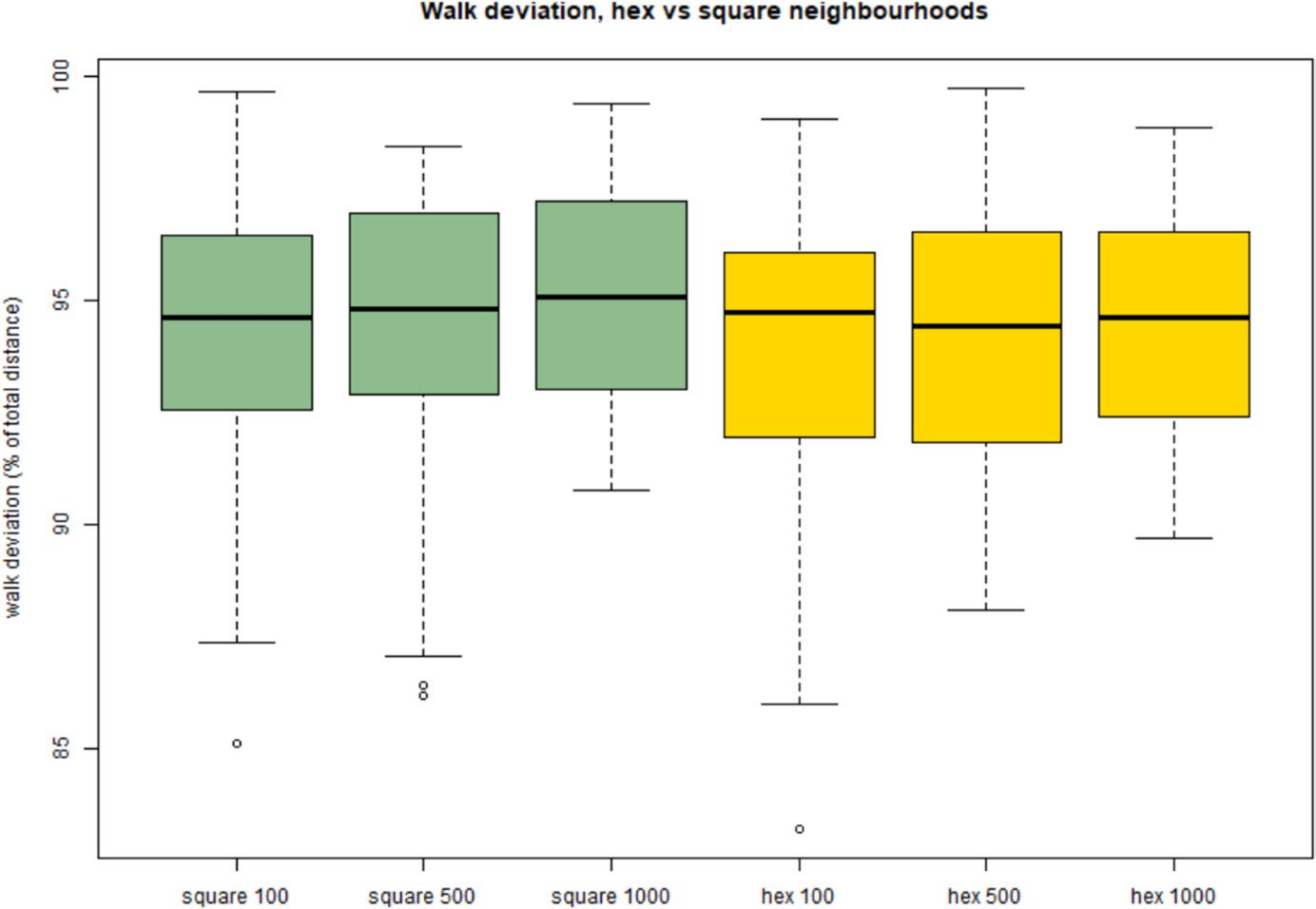
Walk deviation—the difference between total walk distances and resultant distances—for all 6 model runs

At first glance, walks undertaken with hexagonal neighbourhoods (HN) seemed very different to those with square neighbourhoods (SN). In particular, SN walks tended to be longer, even if the straight-line distances from start to finish were broadly comparable (Fig. 8). This is likely to be due to the fact that, following Pythagoras’ Theorem, diagonal distances through the square neighbourhoods are longer than orthogonal distances (neighbourhood size * $\surd 2$) while distances through the hexagonal neighbourhood are the same in any direction (neighbourhood size * 1). Overall, in model runs with the same neighbourhood size, square neighbourhood configurations are likely to produce longer distances overall. Logically, large neighbourhoods also led to longer distances overall (Fig. 8). This was the case both for the resultant distances (the straight-line distance from start to finish) as well as for the total distances (the accumulated distance travelled by all walks).

**Fig. 9:**
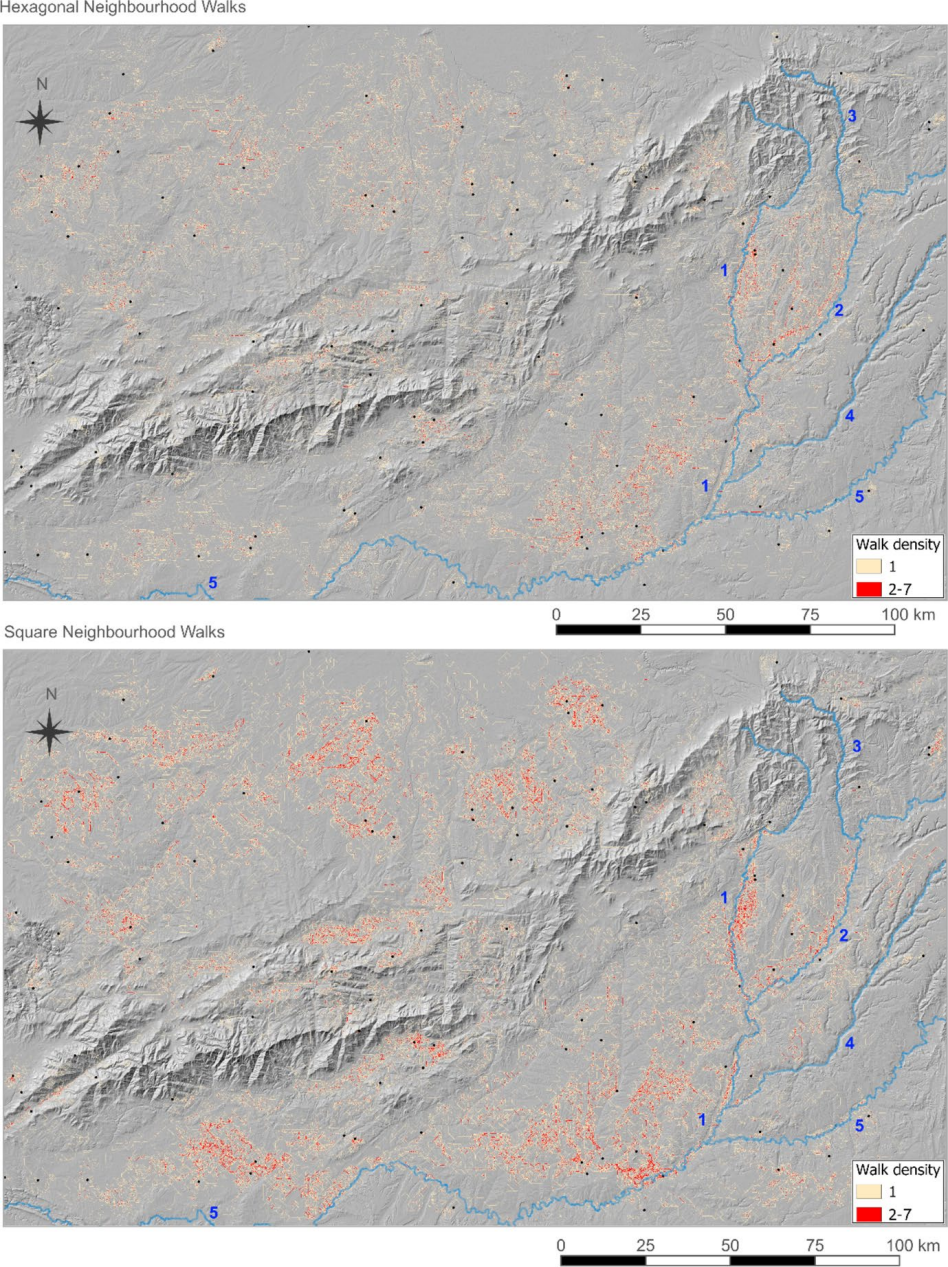
Aggregate results of 100 least cost walks for 1000 m, 500 m and 100 m neighbourhood sizes (cost decision catchments) from randomly determined starting locations (black dots) in the Central Iberian Mountain region. The legend indicates walk density, coloured red where two or more walks cross the same cell. The background map shows terrain slope with darker greys representing more steeply sloping terrain. Numbers indicate major rivers: 1: Jarama; 2: Henares; 3: Sorbe; 4: Tajuña; 5: Tajo

Walk deviation (Fig. 8), expressed as a percentage of total walk distance, was generally greater for square neighbourhoods (green). However, in aggregate, the most-visited locations were similar (Fig. 9). In particular, the river corridors of the Jarama and Henares, to the south of the mountain chain, were particularly favoured dispersion corridors, with many walks concentrated in these areas both in HN and SN modes (Fig. 9).

**Fig. 10:**
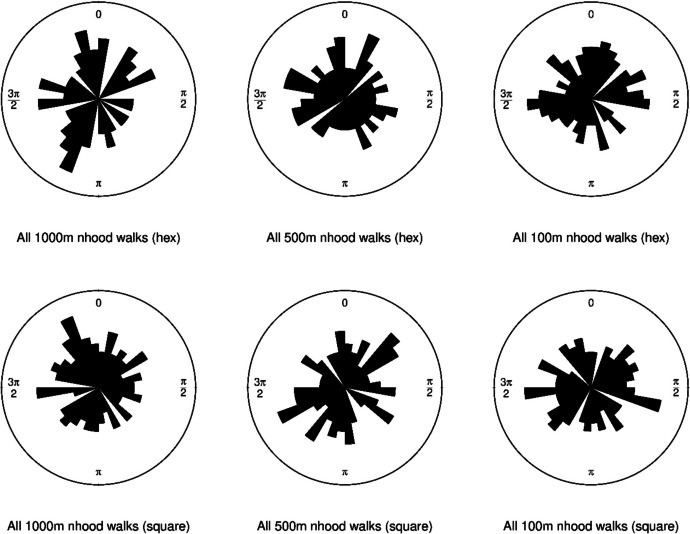
Rose diagrams of resultant azimuth bearings for all 100 walks in each of the 6 simulations

Orientation rose diagrams (Fig. 10) did not suggest preferential orientation for the mean directions of any of the walks. The Rayleigh test suggested no significant deviation from a uniform distribution about the circle for either hexagonal neighbourhoods or square neighbourhoods in any of the 6 cases. However, this test is usually recommended in the case of a unimodal distribution (strongly preferential orientation in one direction), and a bimodal distribution cannot be ruled out. For this reason, the Kuiper test was applied, following Bogdan *et al*. (2002). In only one case, for the 100 m length walks under the hexagonal neighbourhood configuration, was there any suggestion of preferential orientation at the rather weak test threshold of *p* < 0.05 (Kuiper’s test, *N* = 100, Test Statistic = 1.834, 0.025 < *P*-value < 0.05). Generally, a preferential orientation can probably be ruled out. This is perhaps unsurprising, given that starting points for walks were assigned at random. It is likely however, that multiple simulation runs of the model from a single, tightly constricted starting point, such as a cave in difficult to access terrain, would indeed show preferential orientation of access and egress, since cost pathways would be much more closely constrained. This task we leave for future work.

## Discussion and Conclusions

### Implications of the Case Study

There is a notable scarcity of Late Pleistocene archaeological sites in central Iberia, especially for the Early Upper Palaeolithic (see *e.g.* Kehl *et al*., 2018; Wolf *et al*., 2018; Alcaraz-Castaño *et al*., 2021; Sala *et al*., 2024). One possible explanation for this apparent lacuna is that the Central Iberian mountains, probably combined with the Iberian System range, presented a significant barrier to hunter-gatherer groups moving south, especially during harsh environmental conditions, as proposed by the ‘Ebro Frontier’ model (Zilhão, 2021; see above). Though our study can hardly be considered to provide conclusive evidence, this simulation model based on terrain cost does suggest that natural topographic corridors facilitating movement across the mountains would not necessarily have been easy to find. While there are, in the present day, several crossing points that give no difficulty to a determined walker, most are not obvious without prior knowledge, with the possible exception of the broad opening of the valley of the River Alberche between the Gredos and Guadarrama ranges.

While the 100 m neighbourhood walks in the Central Iberia case study were not typically long enough to cross the range completely, the 500 m and 1000 m walks were able to do so, in the event that the modelled agents were able to find a natural crossing point and follow it all the way through. Of the 400 total walks from the 500 m and 1000 m neighbourhood runs, only 5 clearly crossed the range in its entirety (from the southern to northern *meseta* or vice-versa) through there were several partial crossings and many incursions into the slopes from lower ground. If the crossing points were ice-covered, and the easiest valley routes less accessible than today (prior to later river valley incisions), crossings might have been limited to a few travellers with specific local knowledge.

The concentration of least cost pathways in the corridors of the rivers Henares and Jarama may be relevant to different archaeological periods, depending on the extent of transformation undergone during the Quaternary. This result aligns with the observations of Gravel-Miguel & Wren (2018) on the use of multiple LCP variants as a means to identify bottlenecks in the landscape. It seems that the Jarama river, in particular, is one such bottleneck, as also implied by the significant number of Middle and Upper Palaeolithic sites located in this river’s basin and its surrounding area (Alcaraz-Castaño, 2023; Navazo *et al*., 2020; Sala *et al*., 2021, 2024).

Beyond the specific implications for the central Iberia case, the DISPERSCA model has some advantages when compared to traditional least cost analysis. Firstly, a simple LCP is useful only if *both* the origin and destination points are known. The DISPERSCA model, when terrain slope is used as the cost variable determining the agent’s movement at each time step, shows the hypothetical least cost for individuals or groups taking multiple journeys (*e.g.* to access resources) to and from any location. The usefulness of the approach lies in the ability to highlight habitual points of access/egress to sites in very rough terrain, and potentially, most frequently visited areas of the nearby landscape. In this sense, the model allows hunter-gather mobility to be explored in a probabilistic way, as movement around a fitness surface, potentially revealing a range of patterns in the landscape, not only potentially favoured pathways or crossing points, but also barriers or potentially important resource locations like outcrops for lithic raw materials. This can be clearly seen in Figs. 5, 6, and 7, where the green line illustrates the anisotropic cost path that would have been traced between the start and finish points *if both these points were known in advance*. Of course, in our case the finish point was not previously known, it is an artefact of the model—the destination of the least cost walk after 1000 steps. It is worth pausing a moment to reflect on this. DISPERSCA does not aim to find the easiest path between two points, but to discover the pattern of exploratory route-finding given cost (or some other) criteria.

Further, the neighbourhood transition function which guides movement decisions need not necessarily be related solely to cost in the sense of terrain relief, slope, or roughness (Winder *et al*., 2015). For example, it can be used to simulate movement through thickly wooded landscapes, attractiveness to single sources such as water, or, with some small modifications, to model how hunter-gatherer bands might respond to other mobile sources such as game animals.

Finally, the results can be used in aggregate, for example, as in the present case where mean direction (known as the resultant) for each model run was calculated from the azimuths of each of the 1000 individual timesteps that made up each run. This allows orientation analysis to be carried out in search of possible preferential orientation, such as would be expected when moving across landscapes where movement is restricted by gorges or ravines to particular geographical directions.

### Limitations of the Model and Future Work

The model has some limitations. The first of these is that the individual walks undertaken in any square CA neighbourhood of a given cell size are not all of equal length, since, by Pythagoras’ theorem, diagonal movements (*e.g.* from a central to a corner cell) are longer, by a factor of $\surd 2$ than horizontal or vertical displacements. This could be solved by employing a Von Neumann (Rook’s case) neighbourhood, in which the corner cells are ignored, rather than the Moore (Queen’s case) neighbourhood used here, in which they are included. This would have the disadvantage of prohibiting diagonal movement, however, which seems unrealistic. The use of the hexagonal neighbourhood solves the problem more neatly, since all exits from the centre of any hexagonal cell to any adjacent hexagonal cell are the same length. For future applications, we recommend the use of the hexagonal neighbourhood configuration. This aligns with other studies, *e.g.* Mithen & Reed (2002), who used hexagonal cellular automata to model hominin dispersal. This should be balanced against that fact that square neighbourhoods seem to allow for greater walk deviations (potentially indicating more flexible route choice), likely as a result of the higher number of cells in the neighbourhood (8 as opposed to 6).

Secondly, the number of model runs (100 runs of 1000 walks each for 6 different model configurations) was chosen as a reasonable compromise between computation time and the need to adequately demonstrate model behaviour. The chosen walks should be seen as possible outcomes within a potentially infinite set of trajectories, not as the certain result of travel from the starting points under the specified rules. More rigorous analysis of model outcomes for different neighbourhood sizes and larger samples would be a worthwhile future goal. This said, we suspect that despite the potentially infinite variation, walks are likely to converge on those repeatedly visited areas highlighted in Fig. 8. This hypothesis could provide an interesting starting point for a future analysis of long-term model behaviour with large sample sizes.

As previously noted, the use of the model as a dynamic least cost routefinder is purely a matter of convenience, as the easiest and most relatable way to communicate the DISPERSCA model to an archaeological or Quaternary science readership. The transition potential algorithm in the model can easily be used to find spatial costs or opportunities arising from other mapped variables, for example, distance to water, attractiveness of a known site or monument, or presence of predators or prey. The application of the DISPERSCA model to these different contexts is an important priority for future work.

Further, unlike in traditional least cost analysis, which supposes absolute omniscience on the part of the agent, the model assumes no prior knowledge of the terrain at all. While this allows us to model the exploratory movements of pioneers, explorers, or groups operating in unknown territory, supposing ignorance of local travel conditions may be unrealistic in many situations. This can, however, be easily addressed in the DISPERSCA model, by weighting the fitness surface in locations where some prior knowledge is supposed (*e.g.* along known or preferred routes). This would allow for exploration of ‘transit-probability’ landscapes, based on agents’ prior knowledge. The higher the weighting, the lower the uncertainty and the less likely the model would be to deviate from known routes. This non-trivial research objective, however, we leave for future work.

Finally, the DISPERSCA model script is provided at the link in the "Data Availability" section to enable users to replicate our results and experiment with the model themselves. We provide step-by-step instructions on how to download and run the code. However, we recognise that for users unfamiliar with command-line scripting operations there may be a steep learning curve. Providing a wrapper script or user-friendly graphic interface will be a key next step for future work.

## Data Availability

The model scripts, user instructions, and data used for the results discussed in this paper are provided at the following link: https://figshare.com/s/b4ed21c7ab4887e1f814.
